# Intranasal Low-Dose Naltrexone Against Opioid Side Effects: A Preclinical Study

**DOI:** 10.3389/fphar.2020.576624

**Published:** 2020-09-18

**Authors:** Laura Micheli, Lorenzo Di Cesare Mannelli, Elena Lucarini, Carmen Parisio, Alessandra Toti, Bruno Fiorentino, Maria Adele Rigamonti, Laura Calosi, Carla Ghelardini

**Affiliations:** ^1^ Department of Neuroscience, Psychology, Drug Research and Child Health - NEUROFARBA - Pharmacology and Toxicology Section, University of Florence, Florence, Italy; ^2^ Molteni Farmaceutici S.p.A, Florence, Italy; ^3^ Department of Experimental & Clinical Medicine, Section of Anatomy & Histology & Research Unit of Histology & Embryology, University of Florence, Florence, Italy

**Keywords:** intranasal delivery, morphine, naltrexone, opioids side effects, oxycodone

## Abstract

Opioids are broad spectrum analgesics that are an integral part of the therapeutic armamentarium to combat pain in the clinical practice. Unfortunately, together with analgesia, a number of adverse effects can occur such as nausea, vomiting, constipation, gastrointestinal alterations and cognitive impairments. Naltrexone is a competitive antagonist of opioid receptors commonly used to treat opioid addiction; its oral use against agonists side effects is limited by the decrease of opioids-therapeutic efficacy and own adverse effects. The intranasal delivery of naltrexone could offer a quick and effective achievement of CNS based on extracellular mechanisms including perineural and perivascular transport. The aim of the study was to test the efficacy of intranasal low-dose naltrexone in reducing intraperitoneal morphine and oxycodone side effects in rodents. In mice, 1 μg naltrexone intranasally administered 30 min before opioids reduced cognitive impairments and motor alteration induced by 10 mg kg^−1^ morphine and 60 mg kg^−1^ oxycodone in the Passive avoidance and Rota rod tests, respectively. Moreover, naltrexone rebalanced opioid-induced reduction of the intestinal transit and latency of feces expulsion as well as food intake inhibition. Importantly, 1 μg naltrexone instillation did not block analgesia as demonstrated by the Hot plate test. In rats, intranasal naltrexone counteracted the opioid-induced pica phenomenon related to emesis and increased water and palatable food intake. The effects were comparable to that achieved by metoclopramide used as reference drug. Treatments did not influence body weight. Lastly, the safety of the intranasal delivery has been checked by hematoxylin–eosin staining that did not show histological alterations of the nasal cavity. In conclusion, intranasal low-dose naltrexone counteracted morphine and oxycodone induced gastrointestinal and CNS side effects without impairing opioid analgesia. It is a candidate to be a valid clinical strategy deserving deep analysis.

## Introduction

Opioids are the keystone of moderate to severe pain management. Currently, statistics show that about 90% of patients are treated with opioids for chronic pain (Manchikanti et al., 2005; Trescot et al., 2006), and 90% of patients who rely on a pain management center are already on opioid therapy (Manchikanti et al., 2004).

Although some patients can achieve sustained partial pain relief with opioid therapy without intolerable side effects (Portenoy, 1996), many patients are not being treated adequately, for reasons that include concerns about tolerability as well as addiction issues particularly with strong opioids such as morphine and oxycodone (Nicholson, 2003). Nausea, vomiting, and constipation, along with central nervous system side effects, are the principal reasons for discontinuation of opioid analgesic treatment (Harris, 2008). Regarding the CNS, the side effects induced by opioids can be divided into three groups. Reduction of consciousness, sedation, and sleep disturbances are symptoms classified as the first group. The second group includes symptoms such as psychomotor impairment, delirium, hallucinations, dreams, and nightmares affecting the thought process and the ability to react with cognitive impairment. Lastly opioids can exert direct toxic effects on neurons by evoking myoclonus, hyperalgesia, and tolerance, thus representing the third group of CNS side effects (Vella-Brincat and MacLeod, 2007). There is no doubt that successful opioid therapy requires that the benefits of analgesia clearly outweigh treatment-related adverse effects. Multiple approaches have been described to address this problem. The clinical challenge of choosing the best option is enhanced by the lack of studies able to compare multiples therapeutic approaches to manage these problems.

A strategy often used to minimize opioid side effects is the administration of an opioid antagonist either as a co-formulation product or as a second stand-alone drug.

Naltrexone, a competitive antagonist of *μ*, *k*, and *γ* opioid receptors, was synthesized in 1963 and was approved by the FDA in 1984 for the management of alcoholism and opioid addiction (Lee and Elston, 2019). However, the therapeutic benefits are often hampered by the reduction of opioid-induced analgesia and by the appearance of the withdrawal syndrome (Culpepper-Morgan et al., 1992; Liu and Wittbrodt, 2002; Tofil et al., 2006). Moreover, the peripheral antagonist effects may be limited by the oral administration that fails to provide a complete protection since the direct action on the gastro-intestinal tract. The nasal route has received a great deal of attention in recent years as a convenient and reliable method for the systemic delivery of drugs with the aim of dosage reduction and for improving the reaching of target site. Based on these pieces of evidence, the aim of the present study was to evaluate the efficacy of the intranasal injection of naltrexone in reducing the several side effects of morphine and oxycodone administration in rodents. In particular, the effects on gastrointestinal and neurological alterations induced by the two opioids were investigated.

## Materials and Methods

### Animals

For all the experiments described below, male Sprague–Dawley rats and CD-1 mice (Envigo, Varese, Italy) weighing approximately 220–250 g and 20–25 g respectively at the beginning of the experimental procedure were used. Animals were housed in Ce.S.A.L (Centro Stabulazione Animali da Laboratorio, University of Florence) and used at least one week after their arrival. Four rats or ten mice were housed per cage (size 26 × 41 cm^2^); animals were fed with standard laboratory diet and tap water *ad libitum*, kept at 23 ± 1°C with a 12 h light/dark cycle, light at 7 a.m. All animal manipulations were carried out according to the Directive 2010/63/EU of the European parliament and of the European Union council (22 September 2010) on the protection of animals used for scientific purposes. The ethical policy of the University of Florence complies with the Guide for the Care and Use of Laboratory Animals of the US National Institutes of Health (NIH Publication No. 85-23, revised 1996; University of Florence assurance number: A5278-01). Formal approval to conduct the experiments described was obtained from the Italian Ministry of Health (No. 54/2014-B) and from the Animal Subjects Review Board of the University of Florence. Experiments involving animals have been reported according to ARRIVE guidelines (McGrath and Lilley, 2015). All efforts were made to reduce the number of animals used and to minimize their suffering.

### Preparations of Compounds

Naltrexone was solubilized in sterile saline solution and intranasally administered. Morphine (6 and 10 mg kg^−1^; S.A.L.A.R.S., Como, Italy) and oxycodone (45 and 60 mg kg^−1^; Molteni, Florence, Italy) were solubilized in sterile saline solution and intraperitoneally (i.p.) injected 30 after the administration of naltrexone. Control animals received an equal volume of vehicles.

### Protocol for Intranasal Administration

According to Chen et al. (2002) mice or rats after being anesthetized (confirmed by the absence of righting reflex) have been subjected to inoculation with a micropipette; volume 40 μl of a solution of naltrexone 1 μg in 10 min respecting a time of 2 min between one nostril and the other (4 min for the instillation into one nostril, a pause of 2 min and another instillation of 4 min in the other nostril). Control animals were inoculated intranasally with 40 μl of saline solution.

### Hot Plate Test

The method adopted was described by O’Callaghan and Holtzman (1975). Mice were placed inside a stainless-steel container inserted in a precision water bath (KW Mechanical Workshop, Siena, Italy) which was set thermostatically at 52.5 ± 0.1°C. Reaction times (s) were measured with a stopwatch 30 min after administration of the analgesic drugs (morphine or oxycodone). The intranasal administration of naltrexone (1, 3, and 10 μg) was performed 30 min before the administration of the analgesic drugs. The end point used was the licking of the fore or hind paws. Those mice scoring less than 12 s and more than 18 s in the pretest were rejected (30%). An arbitrary cutoff time of 45 s was adopted. Control animals were treated with vehicles

### Passive-Avoidance Test

The test was performed according to the step-through method described by Jarvik and Kopp (1967). The apparatus consists of a two-compartment acrylic box. Alight compartment is connected to a darkened one by a guillotine door. Mice, as soon as they entered into the dark compartment, received a punishing electrical shock (0.5 mA, 1 s). The latency time for entering into the dark compartment was measured in the training session and after 24 h in the retention test. The cut-off for the entry latency allowed in the retention session was 120 s. The deficit in passive avoidance performance was expressed as the difference (in seconds) between retention and training latencies. Naltrexone was administered 30 min before morphine or oxycodone injection in the training session,

### Rota Rod Test

The apparatus consisted of a base platform and a rotating rod (30 cm in length divided into five equal sections by six disks) with a diameter of 3 cm and a non-slippery surface. The rod was placed at a height of 15 cm from the base. Up to five mice were tested simultaneously on the apparatus, with a rod-rotating speed of 16 r.p.m. The motor coordination integrity was assessed on the basis of the time between the moment when the animals were placed on the rotating rod and the moment in which they fall or are removed. The cut-off was 120 s. The animals were trained for two days to remain on the rod for at least 120 s (Kuribara et al., 1977). The intranasal administration of naltrexone (1 μg) was performed 30 min before the administration of the analgesic drugs. The test was performed 30 min after the administration of morphine or oxycodone.

### Evaluation of Intestinal Transit

The experiment has been conducted following the method of Schulz and colleagues using a meal of coal (Schulz et al., 1979). To mice fasted for 24 h with free access to water has been administered by gavage a suspension 1.5 ml of 20% (weight/volume) of carbon in a solution of gum arabic at 5% (weight/volume). The mice, 20 min after receiving the charcoal meal, were sacrificed and the intestine was removed *en bloc*. The transit in the small intestine was calculated for each mouse as the ratio between the distance traversed by the charcoal meal and the total length of the intestine itself.

### Evaluation of Colonic Propulsion

The propulsion of the distal colon has been measured according to the method described by Raffa (1988). 30 min after the administration of morphine or oxycodone, a bead of glass (diameter 5 mm) was inserted in the distal colon of each mouse at 3 cm from the anus. The parameter taken as reference was the time taken by each mouse ejecting the bead. A greater colonic propulsion was represented by a reduction of the time of expulsion.

### Evaluation of Food Intake in Mice

A weighed amount of food (standard laboratory pellets) was given to mice without access to food for 8 h (water was available *ad libitum*), and the weight consumed (evaluated as the difference between the original amount and the food left in the cage, including spillage) was measured 60 min after the injection of opioids, 90 min after the administration of naltrexone.

### Kaolin Preparation and Intake

Kaolin was prepared according to the methods described by Mitchell and colleagues (Mitchell et al., 1976; Mitchell et al., 1977). 99 g of pharmacological grade kaolin (hydrated aluminum silicate, Fisher Scientific Co., Fair Lawn, NJ) was mixed with 1 g of acacia (gum arabic, Fisher Scientific Co., Fair Lawn, NJ), *i.e.*, in a 99:1 ratio, with distilled water to form a thick paste. The paste was rolled on a stainless steel tray and cut into pieces in a shape and size similar to that of regular rat chow pellets. The pellets were placed on steel trays and completely dried at room temperature for 72 h.

### Evaluation of Food, Water and Kaolin Intake in Rats

In rats, a weighed amount of food (standard laboratory pellets), water, and kaolin was given to animals, and the weight consumed (evaluated as the difference between the original amount and the one left in the cages, including spillage) was measured before and 24, 48, 72, and 96 h after treatments. Metoclopramide (5 mg kg^−1^), used as reference drug, was suspended in 1% carboxymethylcellulose sodium salt and orally (p.o.) administered. Control animals were treated with vehicles.

### Body Weight

Body weight was measured before and 24, 48, 72, and 96 h after treatments. Metoclopramide (5 mg kg^−1^, p.o.) was used as reference drug. Control animals were treated with vehicles.

### Histological Analysis

Upon sacrifice, mice were decapitated, and whole heads were decorticated, fixed by immersion in Immunofix (Bio-Optica, Milan, Italy) for 24 h, followed by decalcification in Biodec R demineralizing solution (Bio-Optica), freshly replaced every 2 days, until adequate tissue softening was achieved (approximately 15 days). Then, the anterior part of the skull including the nasal cavities was dissected, embedded in paraffin, and cut transversely in 6 µm-thick sections, which were stained with hematoxylin and eosin (Feldman and Wolfe, 2014).

### Statistical Analysis

Statistical analysis results were expressed as means ± S.E.M of 10 animals per group, performed in two different experimental sets. The analysis of variance was performed by one-way ANOVA. A Bonferroni’s significant difference procedure was used as *post-hoc* comparison. P values of less than 0.05, 0.01, or 0.001 were considered significant. Data were analyzed using the “Origin 9.1” software.

## Results

**Figure 1:**
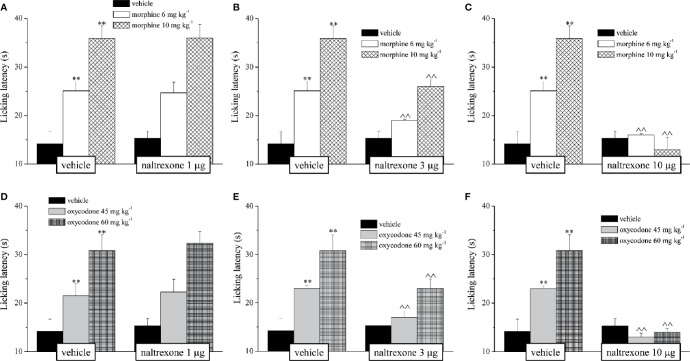
Effect of intranasal administration of naltrexone on morphine and oxycodone analgesia. The response to a thermal non noxious stimulus was evaluating by measuring the licking latency (s) as a pain related behaviour by the Hot plate test. Naltrexone (1, 3 and 10 mg) was intranasally administered 30 min before the intraperitoneal injection of **(A–C)** morphine (6 and 10 mg kg^-1^) or **(D–F)** oxycodone (45 and 60 mg kg^-1^). Behavioural measurements were performed 30 min after opioids treatment. Control animals were treated with vehicles. Each value represents the mean of 10 mice per group, performed in two different experimental set. **P < 0.01 vs vehicle-treated animals; ^^P < 0.01 vs morphine or oxycodone- treated animals.

**Figure 2:**
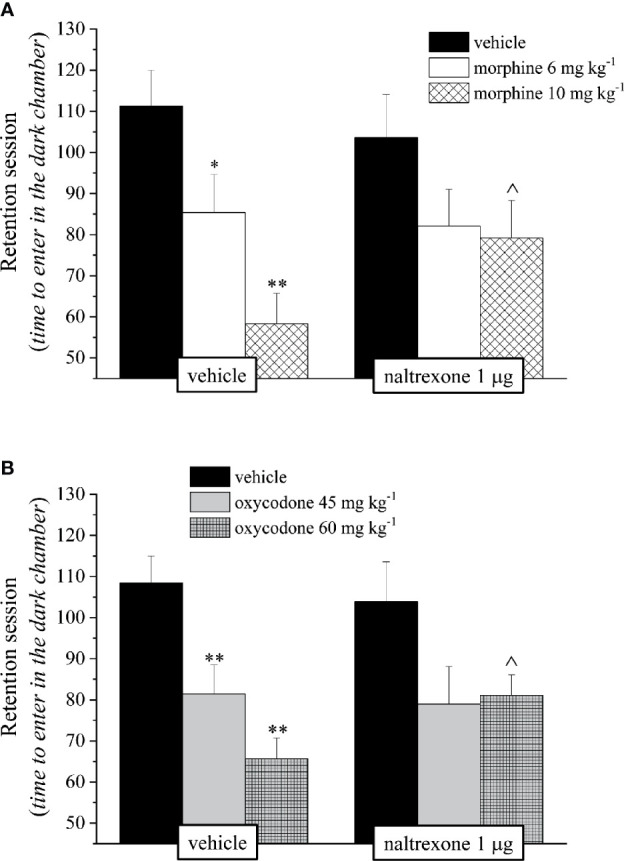
Effect of intranasal administration of naltrexone on morphine and oxycodone induced amnesia in the mouse. Memory impairment was evaluated by the Passive avoidance test. Naltrexone 1 μg was intranasally administered 30 min before the intraperitoneal injection of **(A)** morphine (6 and 10 mg kg^−1^) or **(B)** oxycodone (45 and 60 mg kg^−1^). Behavioral measurements were performed 30 min after opioid treatment. Control animals were treated with vehicles. Each value represents the mean of 10 mice per group, performed in two different experimental sets. *P < 0.05 and **P < 0.01 *vs* vehicle-treated animals; ^P < 0.05 *vs* morphine or oxycodone-treated animals.

Before proceeding with the evaluation of the efficacy of naltrexone in reducing the side effects of morphine and oxycodone, we first individuated the dose of the opioid antagonist that did not interfere with the analgesic effect of the drugs. Naltrexone (1, 3, or 10 μg) was intranasally administered 30 min before morphine (6–10 mg kg^−1^) or oxycodone (45–60 mg kg^−1^); the dose of 1 μg did not reduce the opioid’s analgesia in the mouse Hot plate test, while the higher doses significantly lowered the licking latency time of the mice with respect to the animals treated vehicle (**Figure 1**). Based on these pieces of evidences, during the following tests the dose of naltrexone used was 1 μg/mouse. Intranasal pre-treatment with naltrexone was challenged in reducing the amnesic effect of morphine and oxycodone in the Passive avoidance test (**Figure 2**). 1 μg naltrexone, when administered 30 min before the injection of morphine, was able to significantly antagonize the cognitive deficit induced by morphine at the dose of 10 mg kg 10 mg kg^-1^ i.p. No efficacy was recorded for the lower dose of morphine (**Figure 2A**).

Similarly to what was seen for morphine, also the amnesia induced by oxycodone (60 mg kg^−1^ i.p.) in the mouse Passive avoidance test was reduced in a statistically significant manner by the pre-treatment with naltrexone. On the contrary, the amnesia induced in the same test by oxycodone, at the dose of 45 mg kg^−1^ i.p., was not changed by the administration of naltrexone (**Figure 2B**). Naltrexone administered alone at all times taken into consideration had no effect in the mice Passive avoidance test (**Figure 2**). None of the tested compounds exerts any effect on training latency (Data not shown).

**Figure 3:**
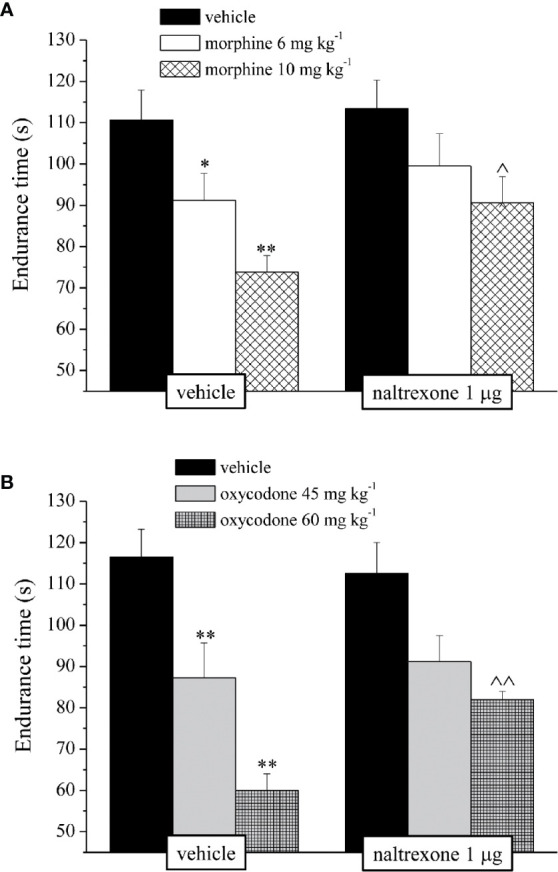
Effect of intranasal administration of naltrexone on morphine and oxycodone induced motor incoordination in the mouse. Motor impairment was evaluated by the Rota rod test. Naltrexone 1 μg was intranasally administered 30 min before the intraperitoneal injection of **(A)** morphine (6 and 10 mg kg^−1^) or **(B)** oxycodone (45 and 60 mg kg^−1^). Behavioral measurements were performed 30 min after opioid treatment. Control animals were treated with vehicles. Each value represents the mean of 10 mice per group, performed in two different experimental sets. *P < 0.05 and **P < 0.01 *vs* vehicle-treated animals; ^P < 0.05 and ^^P < 0.0 *vs* morphine or oxycodone-treated animals.

The reduction of the endurance time exerted by the opioids in a dose-dependent manner in the Rota rod test was counteracted by pre-treatment with naltrexone in both groups treated with 10 mg kg^−1^ morphine or 60 mg kg^−1^ oxycodone (**Figure 3**).

**Figure 4:**
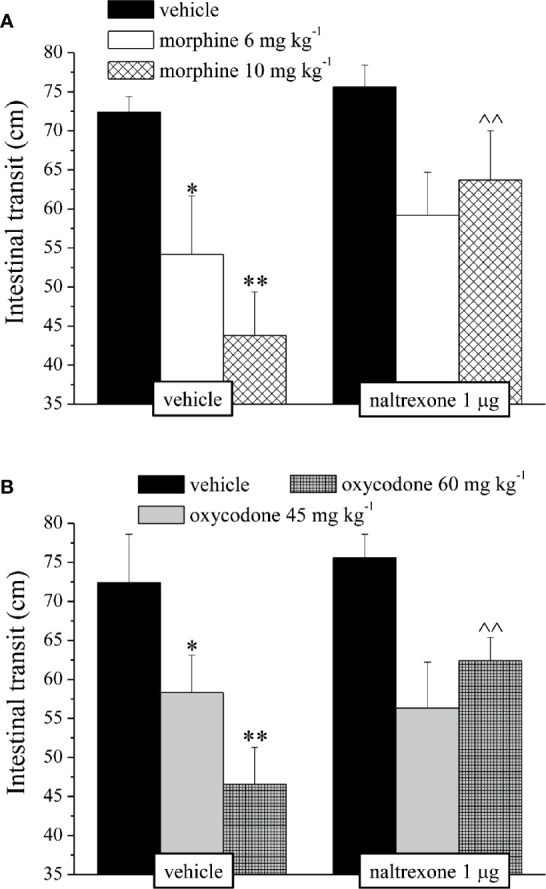
Effect of intranasal administration of naltrexone on morphine and oxycodone induced inhibition of intestinal peristalsis in the mouse. Intestinal transit was expressed as the ratio between the distance traversed by the charcoal meal and the total length of the intestine itself. Naltrexone 1 μg was intranasally administered 30 min before the intraperitoneal injection of **(A)** morphine (6 and 10 mg kg^−1^) or **(B)** oxycodone (45 and 60 mg kg^−1^). Behavioral measurements were performed 30 min after opioid treatment. Control animals were treated with vehicles. Each value represents the mean of 10 mice per group, performed in two different experimental sets. *P < 0.05 and **P < 0.01 *vs* vehicle-treated animals; ^^P < 0.01 *vs* morphine or oxycodone-treated animals.

**Figure 5:**
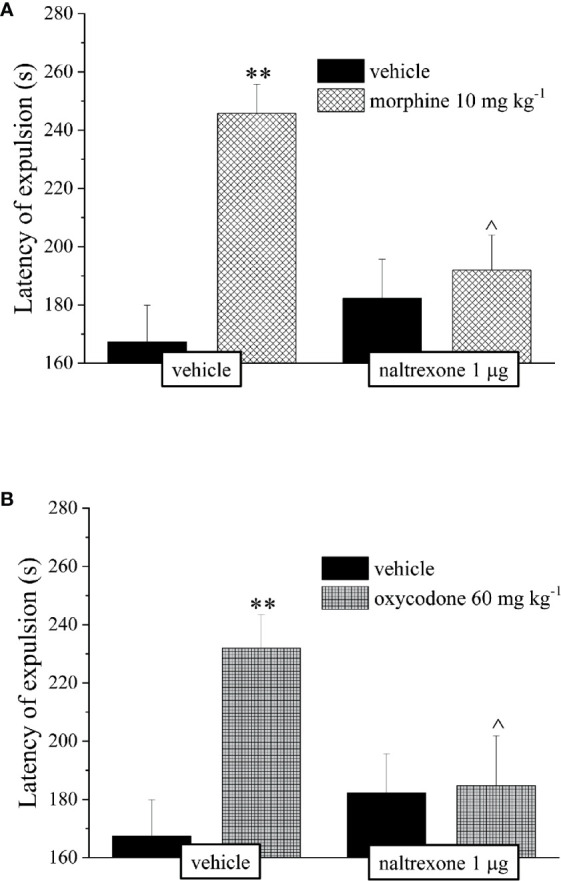
Effect of intranasal administration of naltrexone on morphine and oxycodone induced inhibition of colonic propulsion in the mouse. The propulsion of the distal colon was evaluated by inserting a bead of glass in the distal colon of each animal 30 min after the administration of **(A)** morphine 10 mg kg^−1^ or **(B)** oxycodone 60 mg kg^−1^. Naltrexone 1 μg was intranasally administered 30 min before the intraperitoneal injection of the opioids. Control animals were treated with vehicles. Each value represents the mean of 10 mice per group, performed in two different experimental sets. **P < 0.01 *vs* vehicle-treated animals; ^P < 0.05 *vs* morphine or oxycodone-treated animals.

**Figure 6:**
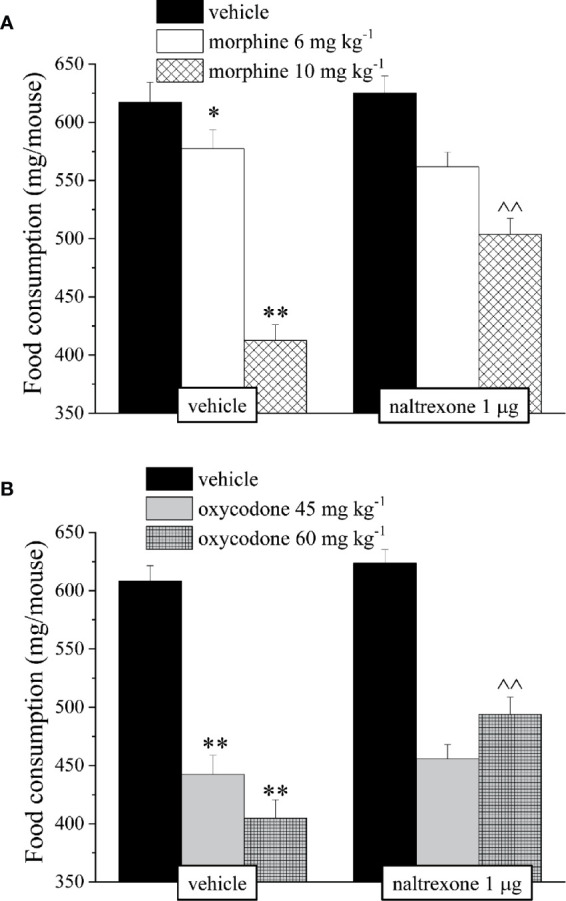
Effect of intranasal administration of naltrexone on morphine and oxycodone induced food intake inhibition in the mouse. Food consumption was expressed as the weight consumed (mg/mouse) during 60 min after the injection of **(A)** morphine (6–10 mg kg^−1^) or **(B)** oxycodone (45–60 mg kg^−1^). Naltrexone 1 μg was intranasally administered 30 min before the intraperitoneal injection of the opioids. Control animals were treated with vehicles. Each value represents the mean of 10 mice per group, performed in two different experimental sets. *P < 0.05 and **P < 0.01 *vs* vehicle-treated animals; ^^P < 0.01 *vs* morphine or oxycodone-treated animals.

It’s well known that opioids also caused gastrointestinal side effects like nausea and vomiting, decrease of appetite and reduction of intestinal transit. In **Figure 4** is represented the antagonism exerted by 1 μg naltrexone on the inhibition of the intestinal peristalsis induced by 10 mg kg^−1^ morphine and 60 mg kg^−1^ oxycodone. Naltrexone, administered 30 min before the opioids, significantly increased the intestinal transit in both groups. Moreover, the higher doses of morphine and oxycodone reduced in mice the colonic propulsion evaluated as the time necessary for the animal to expel the glass bead insert into the distal colon. Similarly to previous results, pre-treatment with naltrexone reverted the increased of morphine and oxycodone regarding the latency of expulsion (**Figure 5**). No effects were recorded when naltrexone was administered alone. Both morphine and oxycodone significantly decreased in a dose-dependent manner the food intake in 8 h food deprived mice (**Figure 6**). The anorectic activity exhibited by the two opioid agonists at the dose respectively of 10 and 60 mg kg^−1^ was reverted by 1 μg naltrexone given 30 min before (**Figure 6**).

The next step of the study was to evaluate the effects of intranasal microdoses of naltrexone in preventing emesis induced by morphine and oxycodone in rats.

Emesis was evaluated measuring the characteristic increased consumption of a non-palatable food, a behavior termed “pica”. Kaolin intake as well as food consumption, water intake, and body weight were evaluated 24, 48, 72, and 96 h after treatment. Metoclopramide (5 mg kg^−1^ p.o.) was used as reference drug.

**Figure 7:**
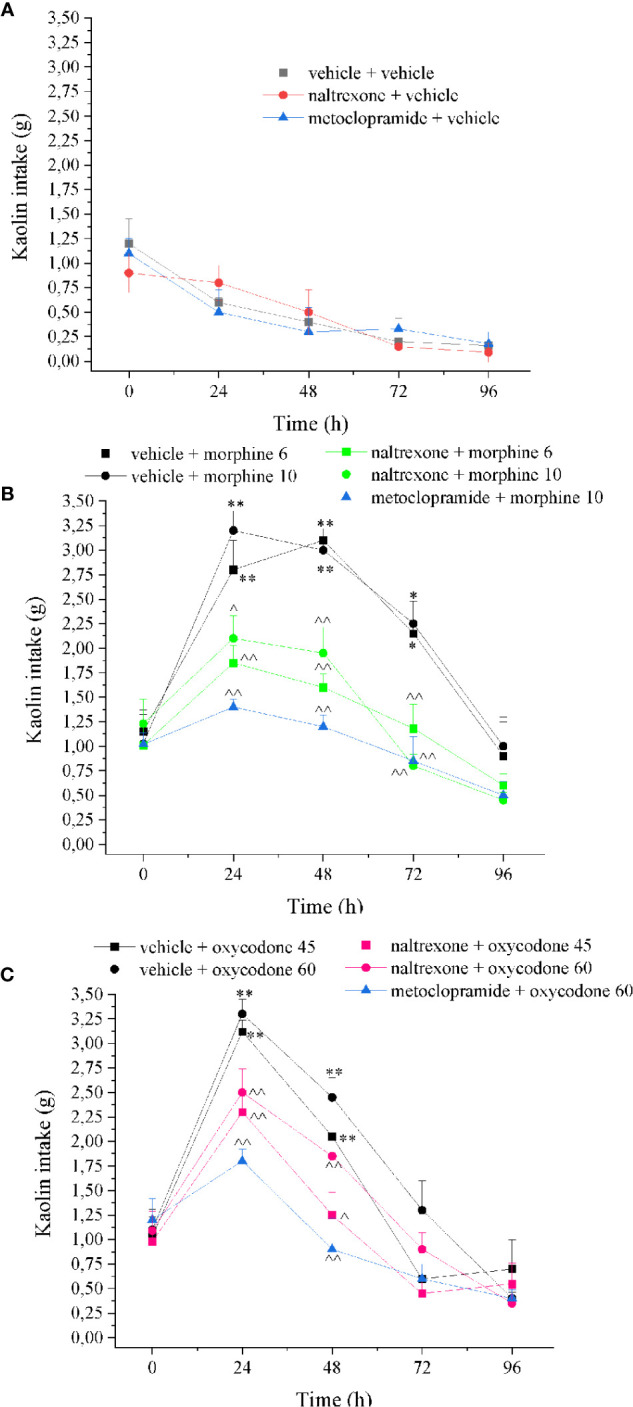
Effect of intranasal administration of naltrexone on morphine and oxycodone induced “pika” phenomenon in the rat. Nausea/emesis related to opioids was evaluated as the increased of the kaolin intake expressed in grams 60 min after the administration of **(A)** metoclopramide (5 mg kg^−1^) or naltrexone 1 μg, **(B)** morphine (6–10 mg kg^−1^) and **(C)** oxycodone (45–60 mg kg^−1^) with the pretreatment with naltrexone 1 μg 30 min before the opioids. Control animals were treated with vehicles. Each value represents the mean of 10 rats per group, performed in two different experimental sets. *P < 0.05 and **P < 0.01 *vs* vehicle-treated animals; ^P < 0.05 and ^^P < 0.01 *vs* morphine or oxycodone-treated animals.

As shown in **Figure 7**, both doses of morphine induced the pica phenomenon related to emesis. Kaolin intake was increased by morphine from 24 to 72 h after administration. A single intranasal dose of naltrexone significantly reduced kaolin intake at all time points. Metoclopramide is a little more efficient without reaching the values of control animals. Similarly, naltrexone prevented the kaolin intake increase induced by oxycodone peaking between 24 and 48 h. Naltrexone and metoclopramide *per se* did not induce effects.

**Figure 8:**
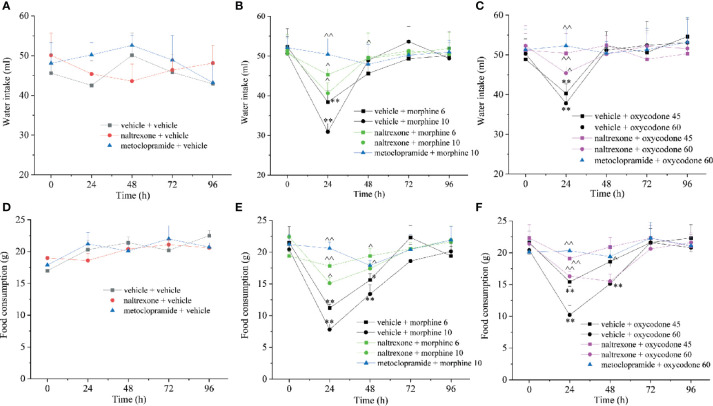
Effect of intranasal administration of naltrexone on morphine and oxycodone reduced food and water intake in the rat. **(A–C)** Water and food **(D–F)** intake were measured up to 96 h after morphine (6–10 mg kg^−1^) or oxycodone (45–60 mg kg^−1^) administration. Metoclopramide (5 mg kg^−1^) or naltrexone (1 μg) was administered 30 min before the opioids. Control animals were treated with vehicles. Each value represents the mean of 10 rats per group, performed in two different experimental sets. *P < 0.05 and **P < 0.01 *vs* vehicle-treated animals; ^P < 0.05 and ^^P < 0.01 *vs* morphine or oxycodone-treated animals.

As previously reported, opioid induced also a decrease of food consumption evaluated at the same time points after treatment (**Figure 8**). The effects of morphine and oxycodone were significant after 24 and 48 h. Naltrexone strongly reduced this effect of opioid agonists; in particular, it fully blocked both morphine and oxycodone when dosed at 6 and 45 mg kg^−1^, respectively. Similarly, the water intake was also altered by the emetic properties of opioid agonists (**Figure 8**). This effect was selectively recorded 24 h after the administration of both morphine and oxycodone. Naltrexone significantly prevented the water intake decrease, reaching the higher efficacy *versus* oxycodone 45 mg kg^−1^ when antagonism allowed for maintaining the values of the control animals. The animal’s body weight was also measured over time remaining unaltered in comparison to controls (**Supplementary Figure 1**). Naltrexone and metoclopramide *per se* did not induce effects in food consumption, water intake, and body weight.

**Figure 9:**
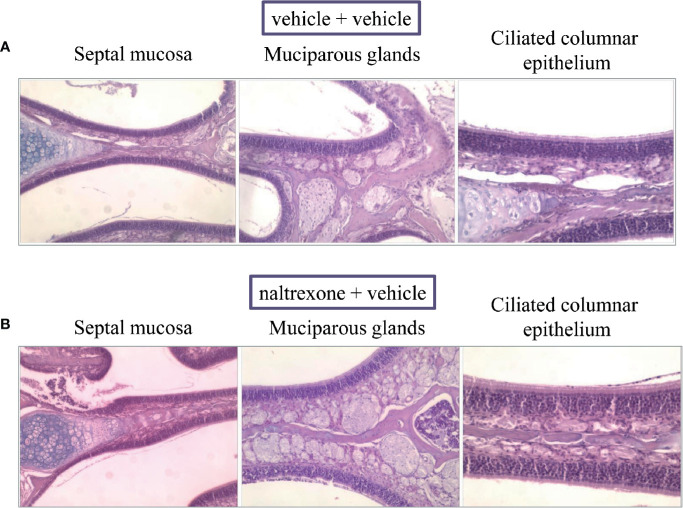
Effect of intranasal administration of naltrexone on mouse nasal cavity. Histological analysis of hematoxylin-and-eosin (HE)-stained cross-sections of whole specimens taken from mice given nasal instillations of **(A)** vehicle or **(B)** naltrexone 24 h before. Septal mucosa, including ciliated surface epithelium, muciparous glands, and stroma, shows a normal appearance in both experimental groups that were analyzed. Images are representative of at least four animals from two independent experiments.

Finally, the safety of the intranasal injection of naltrexone was evaluated by a histological analysis of the nasal cavity 24 h after the administration of 1 µg/mouse. As shown in **Figure 9**, no histological differences were observed between the naltrexone-treated and the vehicle-treated animals. In particular, in both animal groups, the septal and lateral mucosa showed normally appearing ciliated columnar epithelium, muciparous glands and stroma; no signs of mucus hyper-production and secretion were detected in the nasal cavities.

## Discussion

Our study highlighted the efficacy of the intranasal treatment with low-dose naltrexone in reducing morphine and oxycodone side effects. In particular, we demonstrated that naltrexone, intranasally administered, can attenuate gastrointestinal and neurological adverse reactions induced by the administration of the opioids and the efficaciousness of the antagonism used did not impair the analgesic effect of morphine and oxycodone.

The crucial location of pain control is in the CNS. The three major classes of opioid receptors, *μ*, *κ*, and *δ* are situated throughout the CNS and the periphery. However, the major receptor involved in pain modulation is the *μ*-opioid receptor (Shook et al., 1987).

Despite the fact that the analgesics used for pain control remain selective for the *μ*-receptor at normal therapeutic doses, they are not selective for the receptor localization. As mentioned before, in the clinical practice, it is required to maintain opioid analgesia and avoid or minimize the collateral effects that are commonly gastrointestinal and/or neurological. A condition termed “poor responsiveness” is reached when a favorable balance between analgesia and collateral effects cannot be achieved.

Naltrexone, FDA approved for both alcohol and opioid use disorders, is a competitive opioid antagonist with a very high binding affinity for *μ*-opioid receptors (Heading, 2006; Sudakin, 2016). Like other tertiary opioid receptor antagonists, naltrexone is quite lipid soluble and readily crosses the blood brain barrier. It is important to say that this non-selective antagonist block all effects of opioid drugs, including centrally mediated pain relief. A selective antagonism of the effects of opioids on the gut has been achieved with specific dose regimens; however, success at reducing opioid-induced collateral effects has been limited by the likelihood of the antagonists to reverse analgesia or to induce opioid withdrawal (Culpepper-Morgan et al., 1992; Cheskin et al., 1995).

Moreover, the oral administration of naltrexone is not free from its own gastrointestinal symptoms, particularly nausea and vomiting. Other effects are reported such as headaches, skin rashes, decreased mental acuity, depression, anxiety, and loss of energy (Gonzalez and Brogden, 1988). Preclinical studies demonstrated that adding ultra-low doses of oral opioid receptor antagonists (*e.g*., naltrexone or naloxone) to opioids can selectively block the sensitive excitatory mechanism, unveiling a potent inhibitory process and suppressing the associated possible opioid side effects (Crain and Shen, 2000). In human, however, this strategy to add low doses of naloxone (0.6 μg/ml) to morphine in patient-controlled analgesia programs has failed to improve the analgesic properties of the drug and to reduce the opioid side effects (Cepeda et al., 2004). On the other hand, increasing the dose of antagonist reduced the therapeutic benefits of opioids. For these reasons, investigating an alternative but still valuable route to administer naltrexone is a clear necessity.

Since the eighties, intranasal drug administration has gained a growing interest. The intranasal route of administration is characterized to be non-invasive and suitable for a local, systemic, and CNS action. Although, the nasal epithelium appears as a tight barrier, the tightness of the intercellular junctional complex of the nasal mucosa is low due to leaky epithelial tissue, and compared to other mucosae, it is easily accessible (Wolburg et al., 2008; Deli, 2009). In addition, an optimal absorption surface for the drug delivery is provided by the extensive vascularization of the mucosa and the lamina propria (Wengst and Reichl, 2010; Lungare et al., 2016). As mentioned before, the nasal route shows several advantages in comparison to the oral or intravenous administration. It is essentially painless, allows a rapid onset of the therapeutic effect and a higher drug bioavailability due to avoidance of hepatic first-pass metabolism. Moreover, bypassing the BBB, the availability of the drug may potentially increase in the central nervous system (CNS) (Dufes et al., 2003). Furthermore, this route of administration allows for lowering the dosages of the drugs thus reducing their side effects. In this study we firstly evaluated if the intranasal administration of naltrexone could alter the analgesic properties of morphine and oxycodone, and we therefore individuated the dose, 1 μg, that did not interfere with opioid’s analgesia and that was used for all the experiments.

Sedation and decreased cognition are examples of CNS adverse effects associated with opioid use. Commonly they are transient, although some patients require additional therapy to avoid these unwanted effects. Studies have explored various long-term effects of exposure to opioids, and it has been demonstrated that opioid addiction causes disturbances in mood and promotes anxiety, depression, and cognitive impairments (Compton III and Wilson, 2000; Swendsen, 2000).

Nevertheless, the impairment by acute morphine in memory retrieval is also reported. In preclinical evaluations, the effect generated was compared to that induced by scopolamine, suggesting that morphine-induced memory impairment resembles scopolamine-induced deficits (El-Sherbiny et al., 2003). We reproduced in mice memory impairments induced by the acute injection of the opioids, evaluated by the Passive avoidance challenge, and pre-treatment with intranasal naltrexone, 30 min before morphine or oxycodone administration, was able to rebalance the alterations. Moreover, naltrexone was also active in counteracting opioid-induced motor dysfunctions. These results are promising since a recent systematic review highlighted that yet today the effectiveness of opioid antagonists to reduce opioid side effect is still debated, in particular, for the lack of research evaluating potential differences in functional effects among medication types, the route of administration, treatment modality, and length of treatment (Maglione et al., 2020).

Moving to the gastrointestinal problems, although the constipation represents the most common adverse event of long term opioid therapy (40–95%), the adverse events on the GI system result in a more generalized condition called the opioid induced bowel dysfunction (OIBD) (Swegle and Logemann, 2006; Benyamin et al., 2008; Bell et al., 2009). A constellation of symptoms embraces the manifestation of OIBD, like dry mouth, vomiting, bloating abdominal pain, anorexia, gastro-esophageal reflux, hard dry stool, straining to pass bowel movements, and incomplete evacuation (Bell et al., 2009; Brock et al., 2012).

The innervation of the GI system is comprised of two main parts; the enteric nervous system (ENS)— the “brain” of the gut, controlled and regulated by two major plexuses: the myenteric plexus (which controls intestinal motor activity) and the submucosal plexus (which controls secretory and absorptive activity) and the visceral afferents, mediating conscious sensation together with autonomous system nerves to the CNS (Brock et al., 2012). The opioid-induced alterations of the GI tract motility are due to the activation of the opioid receptors that lead to the inhibition of the secretion of several neurotransmitters (Wood and Galligan, 2004). As mentioned before, the most common non-transient side effect of opioids is constipation (Pappagallo, 2001). Opioid-induced constipation is caused by the reduction of the peristaltic movements by the binding of opioids on the *μ*-receptor in the intestines (De Luca and Coupar, 1996) and normally is managed with fibers, linaclotide, and prucalopride, the common treatments used for functional constipation. Recently, specific oral treatments with antagonists directed to the opioid *μ* receptor in the GI tract (PAMORAs) have been made available (*e.g.* naloxegol, naldemedine) (Corsetti and Tack, 2015a; Corsetti and Tack, 2015b). However, surveys have shown that only 46% of patients with constipation originated from opioids achieve desired treatment results >50% of the time (Pappagallo, 2001). A preclinical study also demonstrated that when naldemedine is orally used in a range dose from 10 to 30 mg/kg, a significant delayed reduction of the analgesic effect of morphine was recorded (Kanemasa et al., 2019). Intestinal transit and motility were analyzed in this study after the intraperitoneal injection of morphine and oxycodone, and both were able to alter these parameters at the higher dose tested. Also in these challenges, pre-treatment with intranasal administration of naltrexone rebalanced the variations reducing these common side effects, confirming the efficacy of the intranasal route. The validity of naltrexone against morphine-induced gastrointestinal disorders was previously studied by Webster and colleagues with an open-label study assessing a 12-month safety of a combination of extended-release pellets of morphine sulfate with a sequestered naltrexone core in patients with chronic, moderate to severe pain (Webster et al., 2010).

Of 465 patients receiving one or more doses, 81.3 of patients experienced one or more adverse events, most commonly constipation (31.8%) or nausea (25.2%) assuming that the formulation does not resolve opiate bowel dysfunction or constipation (Webster et al., 2010). Nevertheless, a study conducted by Raffaeli and colleagues demonstrated the efficacy of low-dose naltrexone to prevent several side effects in patients with cancer pain treated with morphine (Raffaeli and Indovina, 2015). However, despite the theoretical basis and the potential advantages of the use of a single opioid antagonist at low doses, so far this approach has not been intensively pursued and needs to be deeply investigated.

Lastly, we evaluated emesis as another common side effect of opioid therapy (Borison and Wang, 1953). This symptom occurs in about one-third of patients treated with morphine, and the incidence severity is roughly in the same ballpark for all opioids (Lehmann, 1997).

The experience of nausea/vomiting may involve multiple receptors (Smith, 2005). Opioid-induced nausea/vomiting (OINV) has not been extensively studied because it is difficult to discriminate it from radiation-induced emesis, chemotherapy-induced nausea/vomiting, or postoperative nausea/vomiting. Despite the fact that the precise mechanisms of OINV are not entirely clear, it can be supposed that multiple and complex tools are implicated including the enhancement of the vestibular sensitivity, a direct effect on the chemoreceptor trigger zone and a delay of gastric emptying. We evaluated emesis in rats measuring the characteristic increased consumption of non-nutritive substances, a behavior termed “pica” (Mitchell et al., 1976) after morphine and oxycodone administration. Kaolin consumption increased, while decreased normal food and water intakes were restored by intranasal pretreatment with naltrexone. Despite the reduction of palatable food intake, the body weight of the animals wasn’t changed by the treatment used. Most probably no effects on weight animals were recorded since we evaluated the effects only up to 96 h after treatments.

Lastly, a histological evaluation of the nasal cavity of the animals 24 h after the administration of naltrexone was conducted to validate the safety of the route of administration. No histological alterations were highlighted on naltrexone-treated animals in comparison to the control group underling the safety of the treatment.

## Conclusion

In conclusion, intranasal low-dose naltrexone was able to counteract morphine and oxycodone induced gastrointestinal and CNS side effects with no action on opioid’s analgesia. These promising results require further in-depth studies for a possible clinical use.

## Data Availability Statement

The raw data supporting the conclusions of this article will be made available by the authors, without undue reservation.

## Ethics Statement

The animal study was reviewed and approved by The Italian Ministry of Health.

## Author Contributions

LM: Investigation, Methodology, data curation, writing—original draft. EL, CP, LCa, and AT: Investigation, software. LCe: Supervision, writing—original draft. BF and MR: Validation. CG: Supervision, conceptualization.

## Funding

This research was funded by the University of Florence and by the Italian Ministry of Instruction, University and Research (MIUR). The authors declare that this study received funding from Molteni farmaceutici S.p.A. The funder was not involved in the study design, collection, analysis, interpretation of data, the writing of this article or the decision to submit it for publication.

## Conflict of Interest

BF and MR are employees of Molteni Farmaceutici S.p.A.

The remaining authors declare that the research was conducted in the absence of any commercial or financial relationships that could be construed as a potential conflict of interest.
