# Supplementary material for: Intranasal Low-Dose Naltrexone Against Opioid Side Effects: A Preclinical Study
Source: Front Pharmacol. 2020 Sep 18;11:576624. doi: 10.3389/fphar.2020.576624 (PMC7531600; doi:10.3389/fphar.2020.576624)
Supplement: Supplementary file 1 [file DataSheet_1.docx]

Supplementary Material
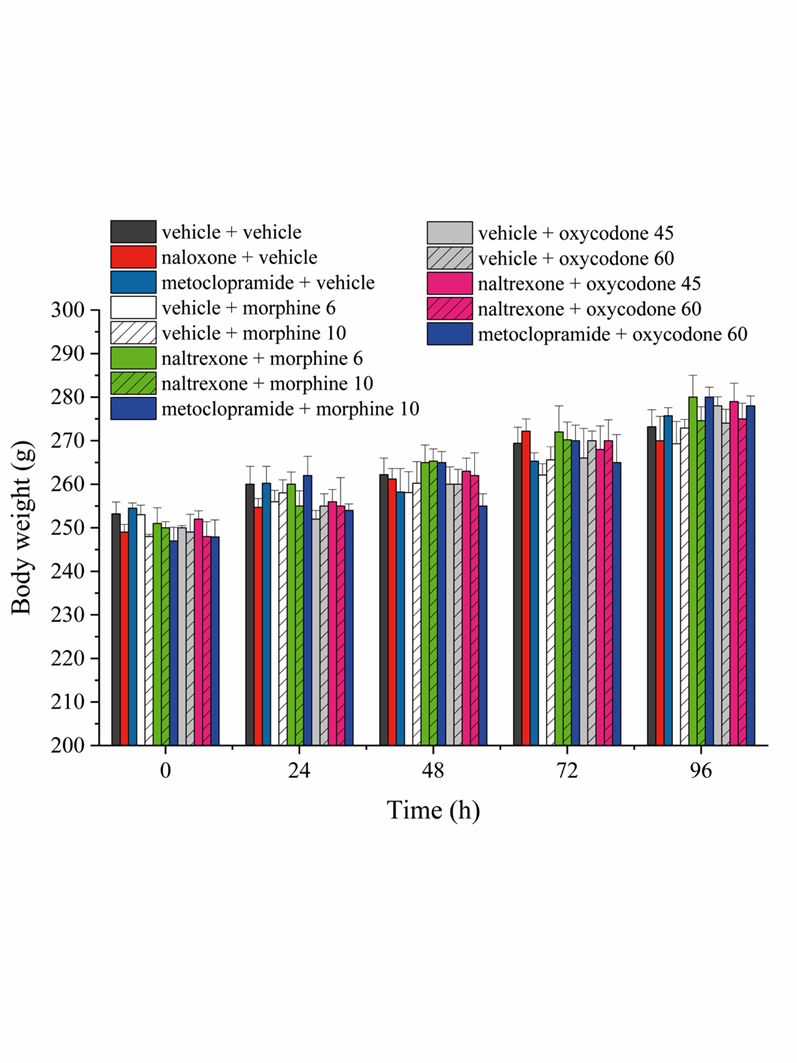


**Supplementary Figure 1.** Effect of intranasal administration of naltrexone on body weight of morphine and oxycodone treated rats. Animal’s body weight was measured up to 96 h after morphine (6 - 10 mg kg^-1^) or oxycodone (45 - 60 mg kg^-1^) administration. Metoclopramide (5 mg kg^-1^) or naltrexone (1 μg) were administered 30 min before the opioids. Control animals were treated with vehicles. Each value represents the mean of 10 rats per group, performed in two different experimental set.
